# Inducible hepatic expression of CREBH mitigates diet-induced obesity, insulin resistance, and hepatic steatosis in mice

**DOI:** 10.1016/j.jbc.2021.100815

**Published:** 2021-05-21

**Authors:** Christopher S. Krumm, Xu Xu, Curtis J. Bare, Corey D. Holman, Sander Kersten, Lukas E. Dow, Ann-Hwee Lee, David E. Cohen

**Affiliations:** 1 Division of Gastroenterology & Hepatology, Joan & Sanford I. Weill Department of Medicine, Weill Cornell Medical College, New York, New York, USA; 2 Nutrition, Metabolism, and Genomics Group, Division of Human Nutrition and Health, Wageningen University, Wageningen, the Netherlands; 3 Division of Hematology & Medical Oncology, Joan & Sanford I. Weill Department of Medicine, Sandra and Edward Meyer Cancer Center, Weill Cornell Medical College, New York, New York, USA; 4 Department of Pathology & Laboratory Medicine, Weill Cornell Medical College, New York, New York, USA

**Keywords:** brown adipose tissue, beige adipose tissue, energy metabolism, insulin resistance, lipid metabolism, nonalcoholic fatty liver disease, obesity, thermogenesis, Ad-GFP, adenovirus encoding GFP, Ad-nuclear CREBH, adenovirus encoding nuclear CREBH, ALT, alanine aminotransferase, ApoA-IV, apolipoprotein A-IV, AST, aspartate aminotransferase, BAT, brown adipose tissue, cDNA, complementary DNA, Cidec, cell death–inducing DFFA-like effector C, Cre, albumin-Cre, CREBH, cyclic AMP-responsive element-binding protein H, ER, endoplasmic reticulum, Fgf, fibroblast growth factor, Fgf21, fibroblast growth factor-21, HFD, high-fat diet, IR, insulin resistance, iWAT, inguinal white adipose tissue, Kiss1, KiSS-1 metastasis suppressor, NAFLD, nonalcoholic fatty liver disease, RER, respiratory exchange ratio, rtTA, reverse tetracycline transactivator, TRE, tetracycline-regulated element, VO_2_, O_2_ consumption, VCO_2_, CO_2_ release, WAT, white adipose tissue

## Abstract

Cyclic AMP-responsive element-binding protein H (CREBH encoded by *Creb3l3*) is a transcription factor that regulates the expression of genes that control lipid and glucose metabolism as well as inflammation. CREBH is upregulated in the liver under conditions of overnutrition, and mice globally lacking the gene (*CREBH*^−/−^) are highly susceptible to diet-induced obesity, insulin resistance, and hepatic steatosis. The net protective effects of CREBH have been attributed in large part to the activities of fibroblast growth factor (Fgf)-21 (Fgf21), a target gene that promotes weight loss, improves glucose homeostasis, and reduces hepatic lipid accumulation. To explore the possibility that activation of the CREBH–Fgf21 axis could ameliorate established effects of high-fat feeding, we generated an inducible transgenic hepatocyte-specific CREBH overexpression mouse model (*Tg-rtTA*). Acute overexpression of CREBH in livers of *Tg-rtTA* mice effectively reversed diet-induced obesity, insulin resistance, and hepatic steatosis. These changes were associated with increased activities of thermogenic brown and beige adipose tissues in *Tg-rtTA* mice, leading to reductions in fat mass, along with enhanced insulin sensitivity and glucose tolerance. Genetically silencing Fgf21 in *Tg-rtTA* mice abrogated the CREBH-mediated reductions in body weight loss, but only partially reversed the observed improvements in glucose metabolism. These findings reveal that the protective effects of CREBH activation may be leveraged to mitigate diet-induced obesity and associated metabolic abnormalities in both Fgf21-dependent and Fgf21-independent pathways.

Cyclic AMP-responsive element-binding protein H (CREBH, encoded by *Creb3l3*) encodes an endoplasmic reticulum (ER) intramembrane-anchored precursor form that requires intramembrane proteolysis at the Golgi apparatus to generate an N-terminal mature fragment that translocates to the nucleus, where it functions as a transcription factor (1, 2, 3). CREBH is transcriptionally controlled by peroxisome proliferator activated receptor α and the glucocorticoid receptor (4). It is activated in liver by fasting, circadian signals, uptake of plasma fatty acids, inflammation, and ER stress to control a multiplicity of genes, including those that regulate apolipoprotein biosynthesis, fatty acid metabolism, lipid droplet formation, and the innate immune response (5). CREBH is induced in mouse liver under conditions of overnutrition, including obesity, insulin resistance (IR), and experimental nonalcoholic fatty liver disease (NAFLD), playing complex regulatory roles in lipid homeostasis (6, 7, 8), hepatic gluconeogenesis (9), clearance of plasma triglycerides (8), and lipid droplet accumulation within hepatocytes (10, 11). Mice with genetic deletion of CREBH (*CREBH*^*−/−*^) exhibit increased susceptibility to hypertriglyceridemia, obesity, IR, and hepatic steatosis in response to either dietary overnutrition or fasting (6, 7, 8). By contrast, transgenic overexpression of CREBH in mice protects against these effects (12, 13). In humans, CREBH mutations have been identified by exome sequencing of patients with severe hypertriglyceridemia (8). On the balance, CREBH upregulation and activation appears to be protective against the metabolic complications of diet-induced obesity in mice.

Activation of CREBH under conditions of chronic overnutrition and fasting leads to the transcriptional upregulation of the hepatokine fibroblast growth factor (Fgf)-21 (Fgf21) (6, 7, 12). Increased circulating Fgf21 exerts metabolic benefits, including weight loss, reduced concentrations of plasma triglycerides, and improved glucose homeostasis (14, 15, 16, 17, 18, 19) that are associated with increased insulin sensitivity and “browning” of white adipose tissue (WAT) (20, 21). These activities of Fgf21 appear to be largely responsible for the beneficial effects of activating CREBH in the setting of overnutrition.

Although CREBH is upregulated in liver and Fgf21 production is increased by obesity, the capacity of the CREBH–Fgf21 axis to mitigate weight gain and obesity-related metabolic disorders is limited under natural conditions. Here, we tested the hypothesis that acute overexpression of CREBH in liver could reverse established obesity, IR, and hepatic steatosis. We generated an inducible transgenic hepatocyte-specific tetracycline-regulated element (TRE)-CRE-reverse tetracycline transactivator (rtTA) overexpression mouse model (*Tg-rtTA*). As expected, doxycycline induced CREBH and Fgf21 expression in a dose-dependent manner (8). Hepatic CREBH overexpression resulted in potent reductions in body weight and adiposity, improvements in glucose homeostasis, and reversal of hepatic steatosis in high-fat diet (HFD)–fed mice. Indicative of both Fgf21-dependent and Fgf21-independent mechanisms, these beneficial effects were largely but not completely reversed upon genetic silencing of Fgf21. Taken together, these findings highlight that the CREBH activation could serve as a potential therapeutic strategy in the management of obesity and associated metabolic disorders.

## Results

### Generation of inducible liver-specific CREBH overexpression mice (*Tg-rtTA*)

Whereas activation of the CREBH–Fgf21 axis protects mice from HFD-induced obesity, IR, and hepatic steatosis (6, 7), the current study was designed to explore whether its activation would reverse these disorders once established. Male mice harbored single copies of the transgenes CAG-Lox-stop-Lox-rtTA3 (rtTA) and albumin-Cre (Cre) in the absence (*Control*) or the presence (*Tg-rtTA*) of a single copy of the transgene CREBH (Fig. S1*A*). Removal of a loxP-flanked polyadenylation signal cassette by Cre-dependent expression driven by the albumin promoter enabled strong cytomegalovirus enhancer, chicken beta-actin promoter and rabbit beta-globin splice acceptor site-driven hepatocyte-specific rtTA expression. Hepatocyte-specific expression of rtTA in turn promoted TRE-mediated CREBH expression in a doxycycline-dependent manner. The mRNA abundance of *Crebh* and associated target genes (*Fgf21* and apolipoprotein A-IV [*ApoA-IV*]) were increased in *Tg-rtTA* mice in a doxycycline-dependent manner (Fig. S1*B*). At a doxycycline concentration of 1 mg/ml in the drinking water, there was no evidence of hepatotoxicity as evidenced by a lack of elevations in plasma alanine aminotransferase (ALT) and aspartate aminotransferase (AST) activities (data not shown) (22), and this concentration was chosen for further studies. Compared with *Control* mice, the mRNA abundance of *Crebh* in livers of *Tg-rtTA* mice was increased 1.8-fold, and protein abundance of nuclear CREBH was increased 16-fold (Fig. S1*C*). Accordingly, the mRNA abundance of the CREBH target genes (*Fgf21* and cell death–inducing DFFA-like effector C (*Cidec*)) were increased in livers of *Tg-rtTA* mice (Fig. S1, *D* and *E*). Although KiSS-1 metastasis suppressor (*Kiss1*) has been reported to be a CREBH target gene that drives Fgf21-independent regulation of glucose metabolism (13), we observed no genotype-dependent differences in hepatic *Kiss1* mRNA abundance (Fig. S1*F*). Plasma Fgf21 was increased by 16-fold in *Tg-rtTA* mice after 3 weeks of doxycycline treatment, and this was sustained at 6 weeks (Fig. S1*G*). These plasma Fgf21 levels in *Tg-rtTA* mice approached those reported following pharmacological administration of Fgf21 to diet-induced obese mice and diabetic rhesus monkeys (17, 23). ApoA-IV was initially undetected in plasma but became abundant after 6 weeks of doxycycline treatment (Fig. S1*H*). ER stress was previously reported to potently induce CREBH in liver and plasma concentrations of Fgf21 (2, 3, 24). However, we did not observe genotypic differences in the mRNA expression of genes that govern ER stress in mice fed chow or HFD (Fig. S2).

### Hepatic overexpression of CREBH reduces fat mass and increases insulin sensitivity by promoting energy expenditure

**Figure 1:**
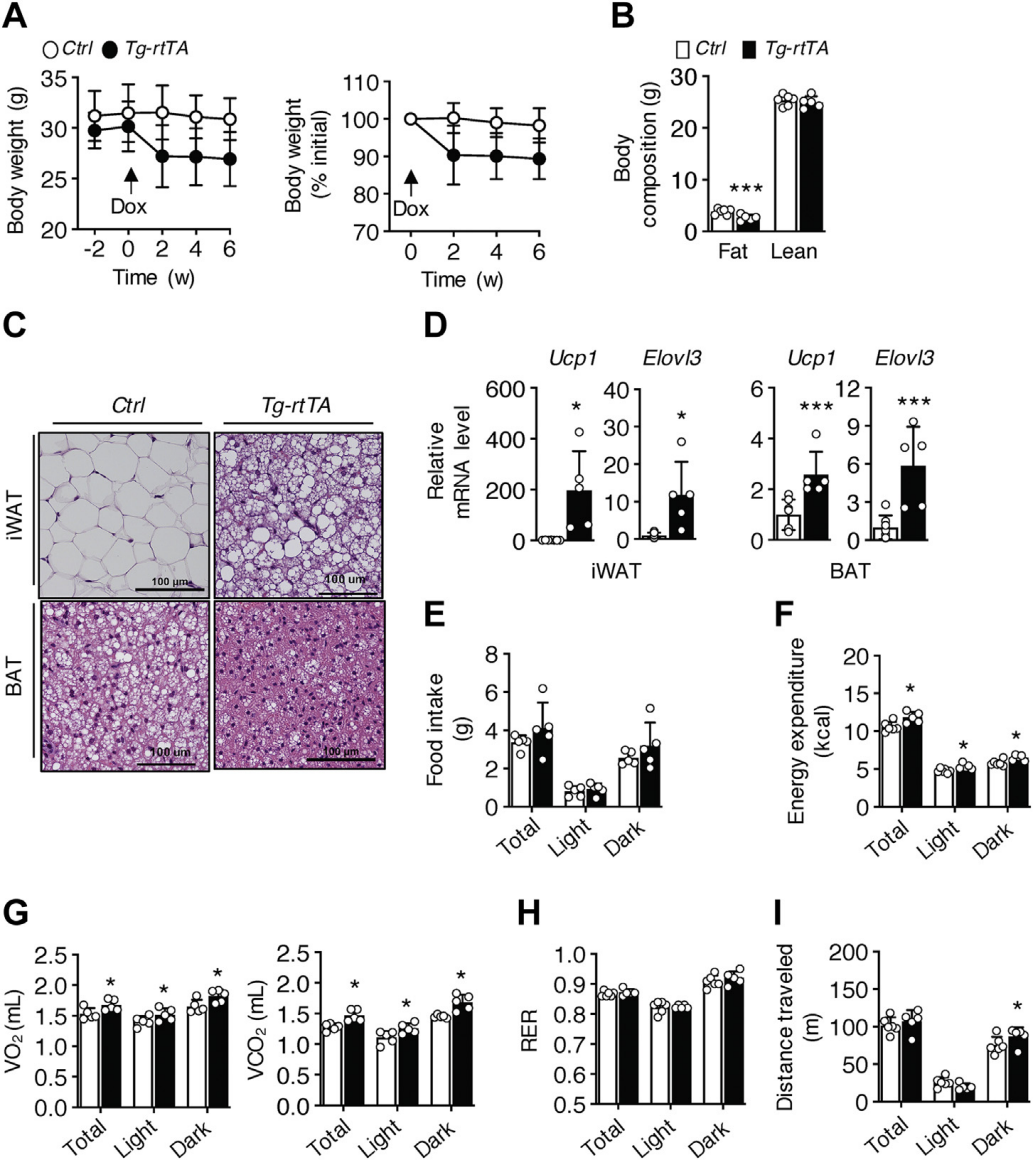
**Reduced body weight and fat mass because of increased energy expenditure in chow-fed *Tg-rtTA mice*.** Six-week-old mice were fed a chow diet for 12 weeks and received doxycycline (Dox; 1 mg/ml) in their drinking water during the last 6 weeks of dietary feeding. *A*, *left panel*, body weights; *right panel*, body weights expressed as a percentage of initial body weight. *Arrow* indicates the start of Dox treatment. *p* < 0.001, effect of genotype. *B*, fat and lean masses of mice treated with Dox for 2 weeks. *C*, representative light microscopic images of H&E-stained inguinal white adipose tissue (iWAT) and brown adipose tissue (BAT) sections. *D*, relative mRNA expression levels of *Ucp1* and *Elovl3* in iWAT and BAT extracts were analyzed by quantitative real-time PCR. *E*–*I*, mice treated with Dox for 2 weeks were individually housed in metabolic cages for the measurement of (*E*) food intake, (*F*) energy expenditure, (*G*) rates of oxygen consumption (VO_2_) and carbon dioxide production (VCO_2_) determined over a period of 24 h, (*H*) values of respiratory exchange ratio (RER) and (*I*) physical activity. *Control*, n = 5 to 8; *Tg-rtTA*, n = 5. Data are means ± SD. ∗*p* < 0.05; ∗∗∗*p* < 0.001; *Control versus Tg-rtTA*. *Elovl3*, ELOVL fatty acid elongase 3; *Tg-rtTA*, inducible transgenic hepatocyte-specific tetracycline-regulated element (TRE)-CRE-reverse tetracycline transactivator; *Ucp1*, uncoupling protein-1.

Considering the potent metabolic effects of Fgf21 therapy on reducing body weight and adiposity (14, 15, 16, 17), we assessed these parameters in *Tg-rtTA* mice. Whereas no genotypic differences in body weight were observed prior to doxycycline treatment, body weights of *Tg-rtTA* mice decreased by 10% within 2 weeks and remained constant for the remainder of the 6-week period (Fig. 1*A*). This reduction in body weight was attributable to a 29% decrease in fat mass relative to *Control* mice (Fig. 1*B*). Histological analysis revealed an increased abundance of adipocytes with multilocular lipid droplets resembling brown adipocytes in inguinal white adipose tissue (iWAT) of *Tg-rtTA* mice, with similar increases in cell densities observed in brown adipose tissue (BAT) (Fig. 1*C*). Activating CREBH in *Tg-rtTA* mice similarly increased expression of the browning markers uncoupling protein-1 and ELOVL fatty acid elongase 3 in both iWAT and BAT (Fig. 1*D*). In the absence of changes in food intake (Fig. 1*E*), *Tg-rtTA* mice exhibited increased total energy expenditure, including both the light and dark cycles (Fig. 1*F*), which reflected increases in values of both O_2_ consumption (VO_2_) and CO_2_ release (VCO_2_) (Fig. 1*G*) without changes in values of the respiratory exchange ratio (RER) (Fig. 1*H*). There were no genotypic differences in total physical activity (Fig. 1*I*). Taken together, these findings were indicative that weight loss could be attributed primarily to increased energy expenditure in *Tg-rtTA* mice owing to thermogenesis.

**Figure 2:**
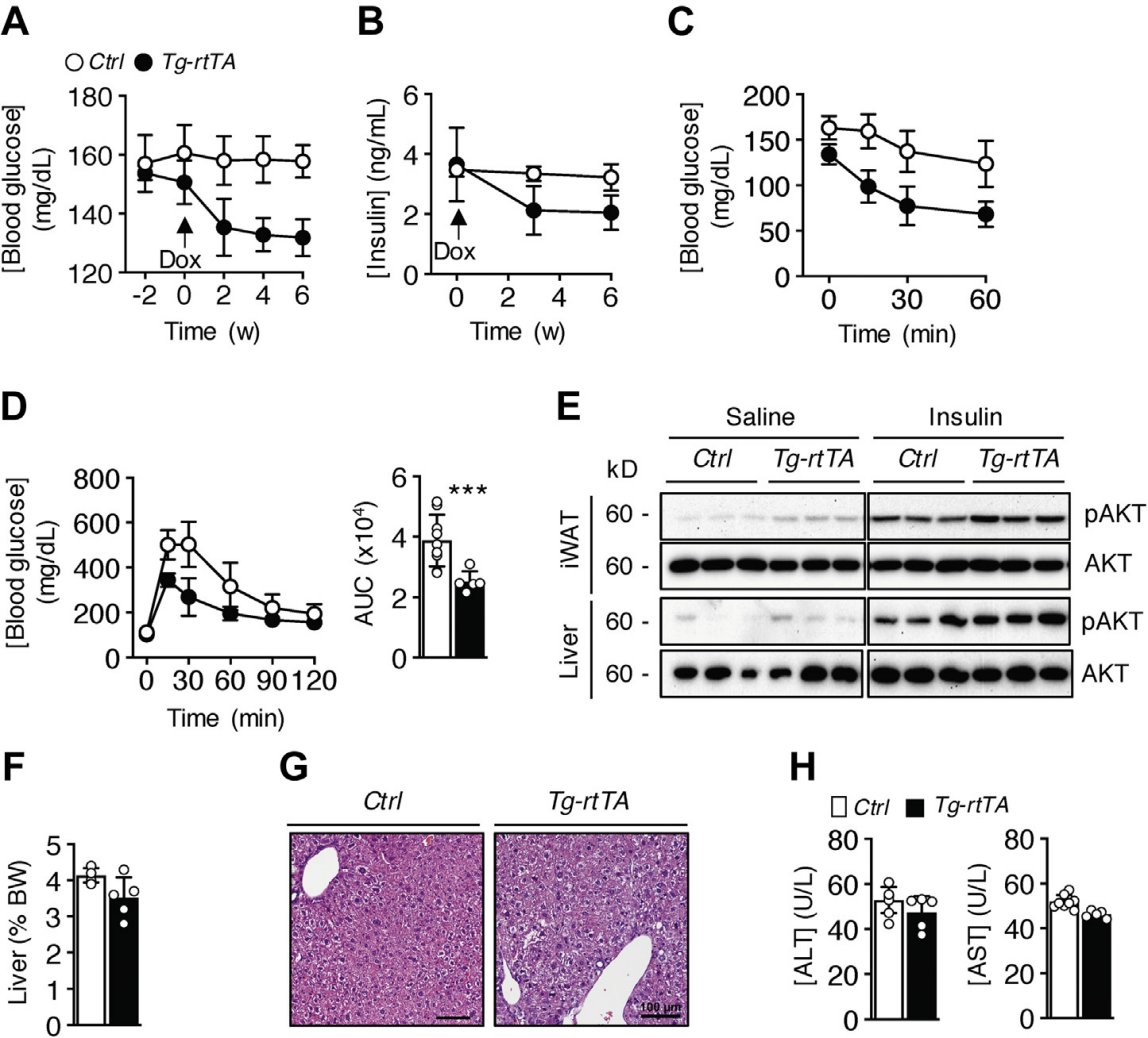
**Increased insulin sensitivity in chow-fed *Tg-rtTA* mice.** Six-week-old mice were fed a chow diet for 12 weeks and received doxycycline (Dox; 1 mg/ml) in their drinking water during the last 6 weeks of dietary feeding. Blood concentrations of (*A*) glucose (*p* < 0.001, effect of genotype and time) and (*B*) insulin (*p* < 0.05, effect of genotype; *p* < 0.001, effect of time). *Arrow* indicates the start of Dox treatment. At 4 and 5 weeks following the start of Dox treatment, respectively, tolerance tests were performed during fasting to (*C*) insulin (*p* < 0.001, effect of genotype and time) and (*D*) glucose (*p* < 0.001, effect of genotype and time). Inset bar plots present values of AUC. *E*, phosphorylated AKT and total AKT protein abundance in liver and inguinal white adipose tissue (iWAT) 10 min following i.p. injection of saline or 1 U/kg insulin. *F*, liver weights of 18-week-old mice. *G*, representative light microscopic images of H&E-stained liver sections. The scale bars represent 100 μm. *H*, plasma activities of ALT and AST. *Control*, n = 5 to 8; *Tg-rtTA*, n = 5. Data are means ± SD. ∗∗∗*p* < 0.001; *Control versus Tg-rtTA*. ALT, alanine aminotransaminase; AST, aspartate aminotransferase; AUC, area under the curve; *Tg-rtTA*, inducible transgenic hepatocyte-specific tetracycline-regulated element (TRE)-CRE-reverse tetracycline transactivator.

Blood glucose concentrations prior to doxycycline treatment were comparable between genotypes (Fig. 2*A*). However, within 2 weeks of doxycycline treatment, blood glucose concentrations dropped by 15.9% in *Tg-rtTA* mice compared with *Control* mice and were sustained up to 6 weeks. Plasma insulin levels prior to doxycycline treatment were also comparable between genotypes and decreased by 42.1% in *Tg-rtTA* mice relative to *Control* mice at 6 weeks (Fig. 2*B*). Following 6 weeks of doxycycline treatment, *Tg-rtTA* mice were sensitized to insulin (Fig. 2*C*) and exhibited increased glucose tolerance compared with *Control* mice (Fig. 2*D*). Accordingly, insulin-stimulated phosphorylated AKT serine 473 protein levels were increased in iWAT and livers of *Tg-rtTA* mice without changes in total AKT protein levels (Fig. 2*E*). These data indicate that the CREBH exerts potent effects on glucose metabolism in *Tg-rtTA* mice. Neither genotypic differences were observed in liver weights (Fig. 2*F*) or histology (Fig. 2*G*) nor were there changes in the plasma activities of ALT and AST (Fig. 2*H*). Enhanced glucose tolerance and insulin responsiveness in *Tg-rtTA* mice were in keeping with the increased metabolic activities of thermogenic brown and beige adipose tissues.

### Hepatic overexpression of CREBH protects mice from diet-induced obesity, IR, and hepatic steatosis

Next, we examined whether the metabolic effects of the CREBH–Fgf21 axis could reverse HFD-induced obesity, IR, and hepatic steatosis in *Tg-rtTA* mice. After HFD feeding for 6 weeks, mice were administered doxycycline in the drinking water in order to activate hepatic CREBH overexpression. The mRNA abundance of *Crebh* in livers of *Tg-rtTA* mice was increased 1.7-fold, and the protein abundance of nuclear CREBH was increased 16-fold (Fig. S3*A*). The mRNA abundance of the CREBH target genes (*Fgf21* and *Cidec*) were also increased in livers of *Tg-rtTA* mice (Fig. S3, *B* and *C*). As observed in chow-fed mice, no genotype-dependent differences in the mRNA abundance of hepatic *Kiss1* were observed (Fig. S3*D*). Plasma Fgf21 was increased by 16-fold in *Tg-rtTA* mice after 3 weeks of doxycycline treatment and was sustained at 6 weeks (Fig. S3*E*). While initially comparable between genotypes, ApoA-IV levels were increased in the plasma of *Tg-rtTA* mice by 6 weeks (Fig. S3*F*).

**Figure 3:**
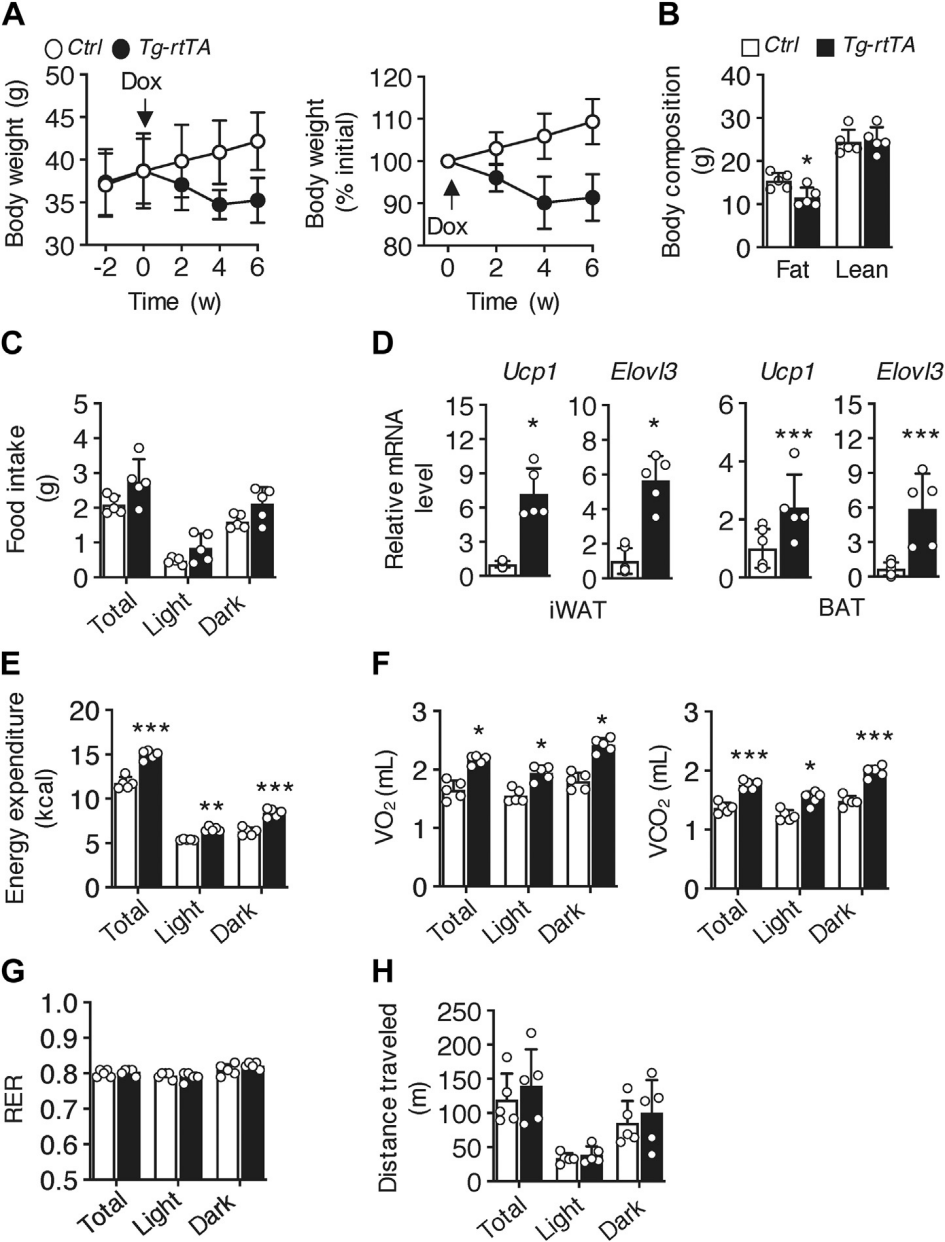
***Tg-rtTA* mice are protected from diet-induced obesity.** Six-week-old mice were fed HFD for 12 weeks and received doxycycline (Dox; 1 mg/ml) in their drinking water during the last 6 weeks of dietary feeding. *A*, *left panel*, body weights; *right panel*, body weights expressed as a percentage of initial body weight. *Arrow* indicates the start of Dox treatment. *p* < 0.001, effect of genotype. *B*, fat and lean masses of mice treated with Dox for 2 weeks. *C*, mice treated with Dox for 2 weeks were individually housed in metabolic cages for the measurement of food intake. *D*, relative mRNA expression levels of *Ucp1* and *Elovl3* in inguinal white adipose tissue (iWAT) and brown adipose tissue (BAT) extracts were analyzed by quantitative real-time PCR. *E*–*H*, mice treated with Dox for 2 weeks were individually housed in metabolic cages for the measurement of (*E*) energy expenditure, (*F*) rates of oxygen consumption (VO_2_) and carbon dioxide production (VCO_2_) determined over a period of 24 h, (*G*) values of respiratory exchange ratio (RER), and (*H*) physical activity. *Control*, n = 5 to 8; *Tg-rtTA*, n = 5. Data are means ± SD. ∗*p* < 0.05; ∗∗*p* < 0.01; ∗∗∗*p* < 0.001; *Control versus Tg-rtTA*. HFD, high-fat diet; *Elovl3*, ELOVL fatty acid elongase 3; *Tg-rtTA*, inducible transgenic hepatocyte-specific tetracycline-regulated element (TRE)-CRE-reverse tetracycline transactivator; *Ucp1*, uncoupling protein-1.

Prior to doxycycline treatment, *Control* and *Tg-rtTA* mice exhibited comparable rates of weight gain upon HFD feeding (Fig. 3*A*). However, within 4 weeks of doxycycline treatment, body weights of *Tg-rtTA* mice decreased by 10%, which could be explained by a 29% reduction in fat mass compared with *Control* mice (Fig. 3*B*) in the absence of changes in food intake (Fig. 3*C*). Expression levels of uncoupling protein-1 and ELOVL fatty acid elongase 3 were increased in both iWAT and BAT of *Tg-rtTA* mice (Fig. 3*D*). *Tg-rtTA* mice exhibited increases in total energy expenditure (Fig. 3*E*) that were attributable to increased values of VO_2_ and VCO_2_ during both the light and dark cycles (Fig. 3*F*) without changes in RER values (Fig. 3*G*). There were also no genotypic-dependent changes observed in physical activity (Fig. 3*H*). As observed in chow-fed mice, these findings suggest that mitigation of HFD–induced obesity in *Tg-rtTA* mice is primarily attributable to increased thermogenesis.

**Figure 4:**
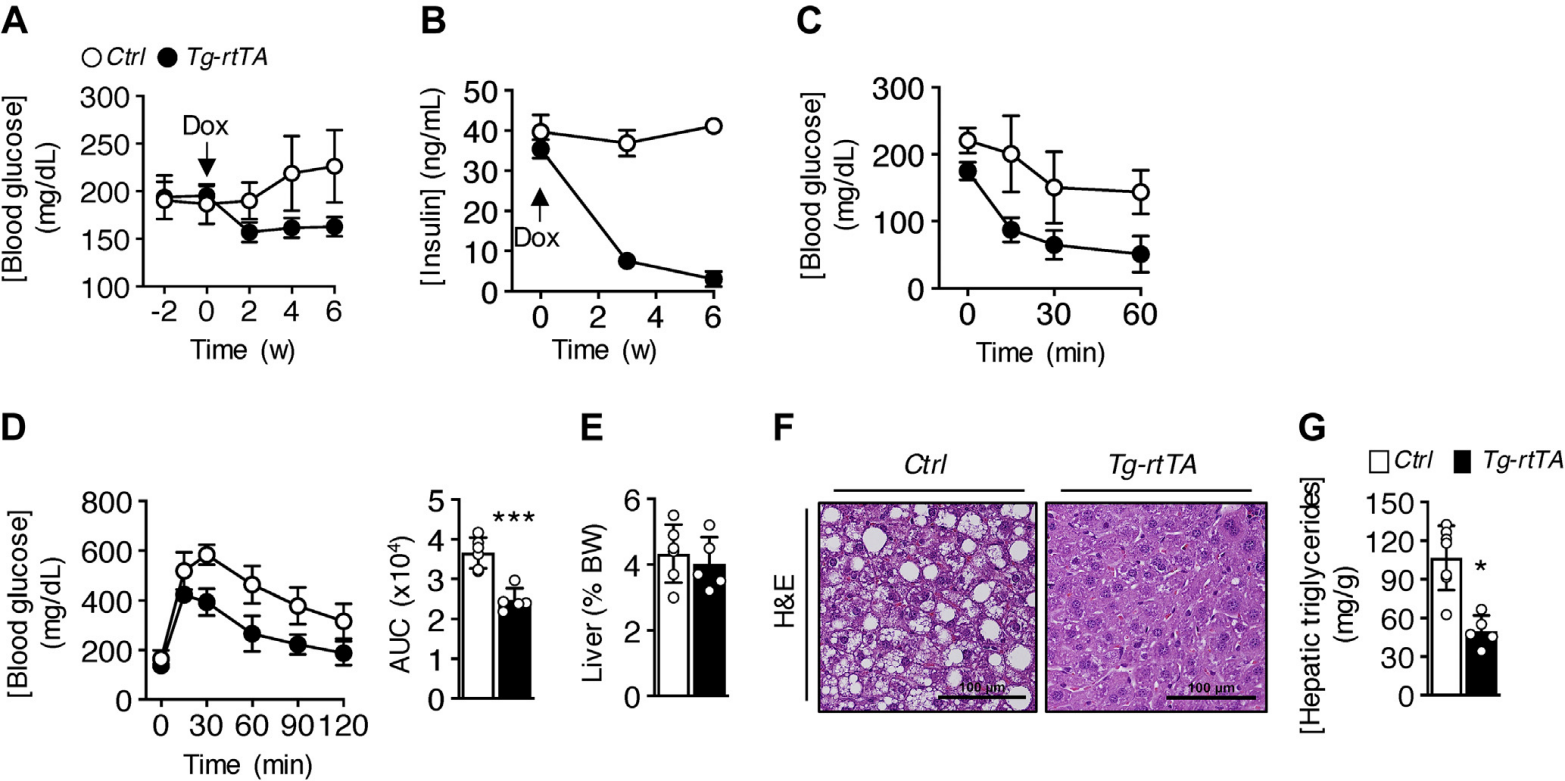
***Tg-rtTA* mice are protected from diet-induced insulin resistance and hepatic steatosis.** Six-week-old mice were fed HFD for 12 weeks and received doxycycline (Dox; 1 mg/ml) in their drinking water during the last 6 weeks of dietary feeding. Blood concentrations of (*A*) glucose (*p* < 0.001, effect of genotype) and (*B*) insulin (*p* < 0.001, effect of genotype and time). *Arrow* indicates the start of Dox treatment. At 4 and 5 weeks following the start of Dox treatment, respectively, tolerance tests were performed to (*C*) insulin (*p* < 0.001, effect of genotype and time) and (*D*) glucose (*p* < 0.001, effect of genotype and time). Inset bar plots present values of AUC. *E*, liver weights, (*F*) representative light microscopic images of H&E-stained liver sections (the scale bars represent 100 μm) and (*G*) hepatic concentrations of triglycerides. *Control*, n = 5 to 8; *Tg-rtTA*, n = 5. Data are presented as mean ± SD. ∗*p* < 0.05; ∗∗∗*p* < 0.001; *Control versus Tg-rtTA*. AUC, area under the curve; HFD, high-fat diet; *Tg-rtTA*, inducible transgenic hepatocyte-specific tetracycline-regulated element (TRE)-CRE-reverse tetracycline transactivator.

Prior to doxycycline treatment during HFD feeding, blood glucose levels were comparable between genotypes. Within 2 weeks of initiating doxycycline treatment, blood glucose levels dropped by 19% in *Tg-rtTA* mice compared with *Control* mice, which were sustained at 6 weeks (Fig. 4*A*). Insulin concentrations were comparable between genotypes before doxycycline treatment but were reduced by 78% in *Tg-rtTA* mice by 3 weeks and sustained at 6 weeks (Fig. 4*B*). Compared with *Control* mice, *Tg-rtTA* mice exhibited increased insulin sensitivity (Fig. 4*C*) and glucose tolerance (Fig. 4*D*).

In previous studies, rodent models of diet-induced hepatic steatosis have been associated with impaired CREBH function (6, 7). Despite comparable liver sizes (Fig. 4*E*), *Tg-rtTA* mice exhibited reduced lipid accumulation by histology (Fig. 4*F*) and hepatic concentrations of triglycerides (Fig. 4*G*). In agreement with our previous studies utilizing *CREBH*^−/−^ mice (6), we did not observe genotypic differences in the mRNA abundance of hepatic genes related to fatty acid oxidation, lipolysis, lipogenesis, gluconeogenesis, inflammation, or fibrosis (Fig. S4). Collectively, these studies suggest that activation of CREBH in liver is sufficient to reverse established resistance to diet-induced obesity, IR, and hepatic steatosis.

### Metabolic improvements in *Tg-rtTA* mice are largely mediated by Fgf21

**Figure 5:**
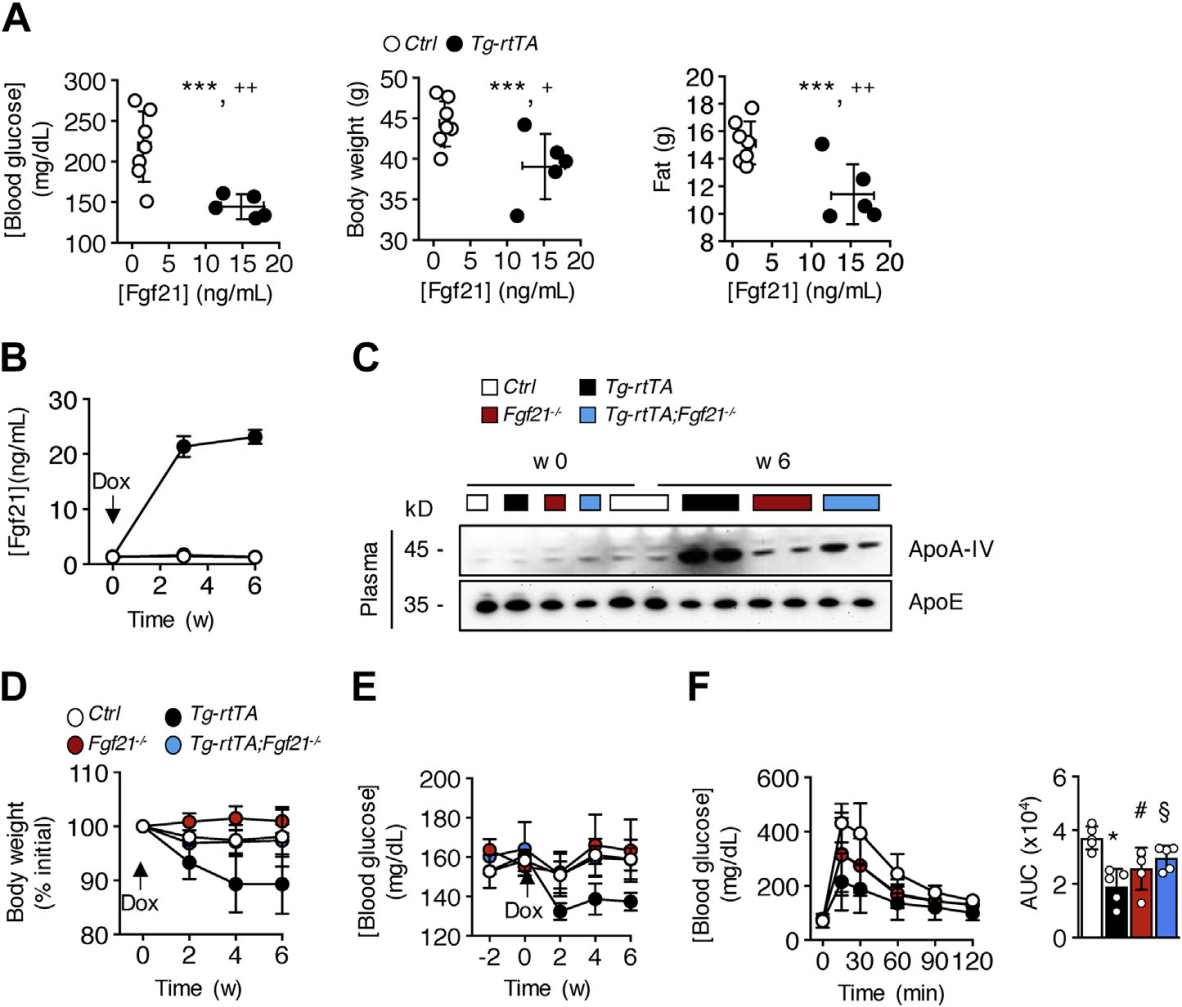
**CREBH-induced metabolic improvements in HFD-fed mice are Fgf21-dependent.** Six-week-old mice were fed HFD for 12 weeks and received doxycycline (Dox; 1 mg/ml) in their drinking water during the last 6 weeks of dietary feeding. *A*, correlative relationships between blood glucose, body weight, and fat mass with plasma concentrations of Fgf21. ∗∗∗*p* < 0.001; *Control versus Tg-rtTA* (*plasma Fgf21*); ^*+*^*p* < 0.05; *Control versus Tg-rtTA* (*body weight*); ^++^*p* < 0.01; *Control versus Tg*-*rtTA* (*blood glucose and fat mass*). *B*–*F*, 6-week-old mice were fed a chow diet for 12 weeks and received Dox (1 mg/ml) in their drinking water during the last 6 weeks of dietary feeding. Plasma concentrations of (*B*) Fgf21 and (*C*) ApoA-IV, with ApoE utilized to control for unequal loading. (*p* < 0.001, effect of genotype; *Tg-rtTA versus Control*, *Fgf21*^−/−^, and *Tg-rtTA;Fgf21*^−/−^). *D*, body weights and (*E*) blood glucose concentrations. *Arrow* indicates the start of Dox treatment (*p* < 0.05, effect of genotype; *Tg-rtTA versus Control*, *Fgf21*^−/−^, or *Tg-rtTA;Fgf21*^−/−^). *F*, glucose tolerance tests were performed at 5 weeks relative to the start of Dox treatment. Inset bar plots present values of AUC. (*p* < 0.05, effect of genotype; *Control versus Tg-rtTA*, *Fgf21*^−/−^, or *Tg-rtTA;Fgf21*^−/−^). *Control*, n = 5 to 8; *Tg-rtTA*, n = 5. *Fgf21*^−/−^, n = 5 and *Tg-rtTA*;*Fgf21*^−/−^, n = 6. Data are means ± SD. ∗*p* < 0.05; *Control versus Tg-rtTA*; ^*#*^*p* < 0.05; *Control versus Fgf21*^−/−^; ^§^*p* < 0.05; *Control versus Tg-rtTA;Fgf21*^−/−^. ApoA-IV, apolipoprotein A-IV; AUC, area under the curve; CREBH, cyclic AMP-responsive element-binding protein; Fgf21, fibroblast growth factor-21; HFD, high-fat diet; *Tg-rtTA*, inducible transgenic hepatocyte-specific tetracycline-regulated element (TRE)-CRE-reverse tetracycline transactivator.

Finally, we tested whether the effects of hepatic CREBH overexpression were primarily attributable to upregulation of Fgf21. Consistent with this possibility, reductions in blood glucose, body weight, and fat mass in *Tg-rtTA* mice were observed in concert with genotypic increases in plasma concentrations of Fgf21 in *Tg-rtTA* mice compared with *Control* mice (Fig. 5*A*). To investigate the mechanistic contribution of Fgf21 in *Tg-rtTA* mice, we crossed *Tg-rtTA* mice to *Fgf21*^−/−^ mice to generate *Tg-rtTA;Fgf21*^−/−^ mice. Prior to doxycycline treatment, comparable plasma concentrations of Fgf21 and ApoA-IV were observed in *Control*, *Tg-rtTA*, *Fgf21*^−/−^, and *Tg-rtTA;Fgf21*^−/−^ mice (Fig. 5*B*). Following 6 weeks of doxycycline treatment, CREBH potently increased plasma Fgf21 levels by 18-fold in *Tg-rtTA* mice relative to *Control*, *Fgf21*^−/−^, and *Tg-rtTA;Fgf21*^−/−^ mice. By contrast, genetically silencing Fgf21 in *Tg-rtTA* mice had no impact on the plasma concentrations of the CREBH target gene ApoA-IV in *Tg-rtTA;Fgf21*^−/−^ mice (Fig. 5*C*).

Ablation of Fgf21 in *Tg-rtTA;Fgf21*^−/−^ mice reversed the body weight loss phenotype in *Tg-rtTA* mice (Fig. 5*D*). Reduced concentrations of blood glucose in *Tg-rtTA* mice were restored to levels comparable to *Control*, *Fgf21*^−/−^, and *Tg-rtTA;Fgf21*^−/−^ mice (Fig. 5*E*). A glucose tolerance test performed after 5 weeks of doxycycline treatment revealed that increased glucose tolerance in *Tg-rtTA* mice remained suppressed following genetic silencing of Fgf21 in *Tg-rtTA;Fgf21*^−/−^ mice (Fig. 5*F*), suggesting Fgf21-independent mechanisms.

To gain insights into liver-specific Fgf21-independent regulatory mechanisms by which CREBH regulates glucose and lipid metabolism, we performed a microarray analysis of mRNA expression in primary cultures of mouse hepatocytes transduced with nuclear CREBH relative to cultured hepatocytes that did not express CREBH. This revealed 1754 significantly upregulated or downregulated genes (Fig. S5). In addition to Fgf21, this analysis yielded several genes with greater fold changes in expression than Fgf21, including those relevant to hepatic lipid, glucose, and energy metabolism: ApoA-IV, solute carrier family 2 (facilitate glucose transporter) member 3 and glycerol kinase 5, as well as hepatocellular carcinoma downregulated mitochondrial carrier protein, which promotes uncoupling of oxidative phosphorylation in liver mitochondria and alleviates hepatic steatosis (25, 26).

## Discussion

Activation of CREBH occurs in response to a multiplicity of stimuli (5), and this liver-enriched transcription factor in turn upregulates genes that restore lipid and glucose homeostasis (11, 27). Among these is Fgf21, which is secreted from the liver and promotes weight loss and improves glucose tolerance and hepatic steatosis (14, 15, 16, 18, 19). Hepatic expression and circulating concentrations of Fgf21 are increased in mice and humans during the onset of obesity and NAFLD (28, 29, 30), suggesting a protective mechanism that is ultimately overwhelmed by sustained overnutrition.

*CREBH*^−/−^ mice are susceptible to hepatic steatosis, at least in part due to increased mobilization of adipose tissue lipolysis, leading to excessive hepatic uptake of plasma fatty acids (6). These effects were reversed upon administration of an Fgf21 recombinant adenovirus (6). Exogenous Fgf21 therapy has also corrected IR and hepatic steatosis in diet-induced obese and *ob/ob* mice as well as diabetic rhesus monkeys (16, 17, 23). Indeed, overexpression of CREBH either genetically or by infection with CREBH adenovirus protected mice from diet-induced obesity through Fgf21-dependent mechanisms (7, 13). Our findings build upon the existing literature by demonstrating that activation of the CREBH axis mitigates these diet-induced abnormalities once established.

Activation of CREBH led to the reduction in lipid droplets in the livers of *Tg-rtTA* mice within an established model of obesity, IR, and hepatic steatosis. This was most likely attributable to the mobilization of triglycerides from lipid droplets required for the assembly and secretion of very low-density lipoprotein particles (27). Under conditions of excessive hepatic triglyceride accumulation including hepatic steatosis, these processes have been shown to be mediated through CREBH by upregulation of ApoA-IV (11, 27). In the setting of overnutrition, upregulation of the CREBH target gene Cidec promotes lipid droplet growth and triglyceride accumulation in the liver (10). However, we did not observe increases in Cidec expression in livers of *Tg-rtTA* mice compared with HFD-fed *Control* mice. These findings suggest that the overall metabolic effects of Fgf21 blunted the upregulation of Cidec by CREBH and thereby contributed to the depletion of lipid droplets.

In mice with diet-induced obesity, a single dose of recombinant Fgf21 is sufficient to improve insulin sensitivity and glucose disposal, and chronic administration promotes body weight loss and reduced adiposity (16, 23). In agreement with these findings and our previous studies (6), we identified strong correlations between plasma Fgf21, and body weight, blood glucose, and adiposity in HFD-fed mice. A mechanistic role for Fgf21 was evidenced by the absence of a CREBH-mediated weight loss effect in Fgf21-deficient *Tg-rtTA;Fgf21*^−/−^ mice. These findings are in agreement with results reported for whole-body transgenic CREBH overexpression in *Fgf21*^−/−^ mice (13). However, improvements in glucose tolerance in *Tg-rtTA* mice were only partially negated in *Tg-rtTA;Fgf21*^−/−^ mice, raising the possibility that CREBH may regulate additional yet-to-be defined factor(s) regulating glucose homeostasis. These findings differ from those in HFD-fed CREBH transgenic mice that expressed CREBH constitutively in the liver, which did not exhibit genotypic differences in glucose disposal (13). It remains to be determined how inducible expression of CREBH in *Tg-rtTA* mice regulates glucose homeostasis differently from constitutively expressed CREBH in CREBH transgenic mice.

By using a doxycycline-inducible system to acutely activate hepatic CREBH in the setting of established obesity, IR, and hepatic steatosis, we have provided new evidence that CREBH-mediated regulation may reveal therapeutic targets for the management of obesity and related metabolic disorders. Our studies suggest that reversal of blood glucose, body weight, and fat mass in established obesity, IR, and hepatic steatosis is, in large part, attributable to Fgf21 in *Tg-rtTA* mice. These observations could be of clinical relevance in obesity-related metabolic disorders, especially because multiple nonsynonymous mutations in CREBH have been reported in human patients with extreme hypertriglyceridemia (8). In contrast to our study of acute and liver-specific CREBH overexpression, Satoh *et al*. (13) studied the influence of whole-body transgenic CREBH overexpression in the development of obesity in response to overnutrition. These findings led them to identify Kiss1 as a novel CREBH transcriptional target partially responsible for driving Fgf21-independent effects on glucose homeostasis in their model. However, we did not detect changes in *Kiss1* mRNA abundance in livers of *Tg-rtTA* mice. Notwithstanding, our results do not exclude the possibility of Fgf21-independent effects of CREBH overexpression in liver.

In addition to identifying significant upregulation of Fgf21 in a microarray analysis of primary cultures of mouse hepatocytes transduced to overexpress nuclear CREBH using recombinant adenovirus, several other genes exhibited greater fold changes than Fgf21 with relevance to hepatic glucose uptake, energy, and lipid metabolism including solute carrier family 2 (facilitate glucose transporter) member 3, glycerol kinase 5, hepatocellular carcinoma downregulated mitochondrial carrier protein, and *ApoA-IV*. In this connection, mice with genetic disruption of ApoA-IV (*ApoA-IV*^*−/−*^) exhibited hyperglycemia and increased susceptibility to diet-induced glucose intolerance. Conversely, pharmacological administration of exogenous ApoA-IV reversed these effects in *ApoA-IV*^*−/−*^ and diabetic KKA_γ_ mice (31). In addition, the microarray analysis revealed two gene sets relevant to lipoprotein metabolism, lipid mobilization, and transport, which were driven by CREBH (32). Genes common to these data and our data that may mediate Fgf21-independent metabolic regulation include major facilitator superfamily domain containing 2A, succinate-CoA ligase GDP/ADP-forming subunit α, and adenylate kinase 2.

Fgf21 administration to rodents increases browning of WAT and BAT, leading to increased energy expenditure and reduced body weight (33). However, the quantitative contribution of thermogenesis to Fgf21-dependent effects on improving body weight, as well as circulating glucose and lipids, is incompletely understood, with evidence that these effects may be driven by thermogenic-dependent and thermogenic-independent mechanisms in beige adipose tissue and BAT (34, 35, 36). Our observations strongly suggest that activating CREBH in the setting of established obesity, IR, and hepatic steatosis promotes weight loss and metabolic improvements by mechanisms that are at least, in part, attributable to increases in thermogenesis.

NAFLD is a common and important comorbidity of obesity with very limited treatment options (37). Fgf21 analogs are currently under development and show promise as potential agents (38). In this study, a hepatocyte-specific transgenic mouse model with inducible doxycycline-dependent CREBH overexpression enabled the demonstration of therapeutic benefits of activating CREBH in experimental NAFLD. Mechanistically, these effects were linked closely to CREBH-mediated transcriptional activation of the hormone Fgf21 but leave open the possibility of additional Fgf21-independent mechanisms. Considering this and the inherent technical challenges of administering protein-based therapies, alternative pharmacologic strategies toward activating CREBH could prove valuable in the management of obesity-associated metabolic disorders.

## Experimental procedures

### Animals and diets

*Tg-rtTA* mice were generated on a mixed C57BL/6;C3H background at the Rodent Genetic Engineering Core of New York University Langone Health (Fig. S1*A*). Transgenic mice expressing rtTA protein containing an upstream loxP-flanked polyadenylation signal for tissue-specific TRE transgene induction using Cre-Lox technology have been previously described (B6.Cg-Gt(ROSA)26Sortm1(CAG-rtTA3)Slowe/LdowJ) (22). These mice were crossed with transgenic mice expressing Cre recombinase driven by the albumin promoter (B6.Cg-Tg(Alb-cre)21Mgn/J; Jackson Laboratory) to generate hepatocyte-specific rtTA mice (*Control*). An Frt-mediated gene targeting system was modified within the Col1A1 locus in mouse embryonic stem cells to generate transgenic TRE-CREBH (*Tg*) mice (39). *Tg* mice were then crossed to *Control* mice to generate *Tg-rtTA* mice. To generate *Tg-rtTA* and Fgf21 knockout (*Tg-rtTA;Fgf21*^−/−^) mice, *Tg-rtTA* mice were crossed to *Fgf21*^*loxp*^ (B6.129S6(SJL)-Fgf21tm1.2Djm/J; Jackson Laboratory) mice. Doxycycline (Alfa Aesar) treatment in the drinking water enabled robust hepatocyte-specific CREBH overexpression in *Tg-rtTA* and *Tg-rtTA;Fgf21*^−/−^ mice. Duplex PCR was performed to distinguish between wildtype and flox alleles using the following primers for transgenic CREBH (forward: TTGACCTCCTGTTTGATCGGCA; reverse: TCCTCAGAGATGCCACTGTCAC) or rtTA (forward: GTTCGGCTTCTGGCGTGTGA; reverse: CGCTTGTTCTTCACGTGCGA; loxP-flanked polyadenylation signal: AAAAACCTCCCACACCTCCC). The presence or the absence of Cre recombinase and Fgf21 was determined by PCR analysis using primers specified by the Jackson Laboratory. For experiments, *Control* mice harbored one copy of the transgenes rtTA and Cre recombinase. *Tg-rtTA* mice harbored one copy of the transgenes CREBH, rtTA, and Cre. Mice were housed in a barrier facility on a 12-h light/dark cycle. Six-week-old male mice were fed a chow diet (Picolab Rodent Diet 20; Lab Diet) or a HFD (60% calories from fat; Research Diets). Unless otherwise specified, mice were studied in the fed state. Tissues were harvested and immediately snap frozen in liquid nitrogen and stored at −80 °C. Animal use and euthanasia protocols were performed using approved guidelines by Weill Cornell Medical College.

### Analytical techniques

Lipids were extracted from liver tissue using chloroform:methanol mixture (2:1 v/v) (40). Enzymatic assay kits were used to measure hepatic and plasma concentrations of triglyceride (Wako Diagnostics). Plasma AST and ALT activities were measured by an enzymatic assay kit (Thermo Fisher Scientific). Plasma insulin and Fgf21 concentrations were determined using commercially available mouse ELISA kits from Crystal Chem and R&D Systems, respectively, according to the manufacturer's specification. Protein concentrations were determined using a bicinchoninic acid assay (Thermo Fisher Scientific).

### Histopathology

Liver, iWAT, and BAT samples were fixed in 10% formalin. Liver samples were sectioned and stained with H&E by the Laboratory of Comparative Pathology at the Center of Comparative Medicine & Pathology (Memorial Sloan Kettering Cancer Center). Images were captured using an Eclipse Ti microscope (Nikon).

### RNA extraction and analysis of gene expression

Total RNA was extracted from mouse liver, iWAT, and BAT using QIAzol lysis reagent (Qiagen) and used to synthesize complementary DNA (cDNA) with a High-Capacity cDNA Reverse Transcription Kit (Applied Biosystems). Gene expression was analyzed with quantitative real-time PCR assays using Power SYBR Green Mix (Applied Biosystems). Real-time PCR assays were performed in duplicate with a total reaction volume of 25 μl containing 500 nM concentrations of each primer and cDNA (25 ng). mRNA expression levels were normalized to the housekeeping gene Actβ. Table S1 provides nucleotide sequences of primers.

### Immunoblot analysis

Tissue extracts were prepared using radioimmunoprecipitation assay buffer (10 mM Tris–HCl [pH 7.4], 150 mM NaCl, 1% Nonidet P-40, 1 mM phenylmethylsulfonyl fluoride, 1 mM EDTA, 1 mM NaF, 0.25% sodium deoxycholate, 10% glycerol, and supplemented with protease and phosphatase inhibitors; Thermo Fisher Scientific). Proteins were separated on 10 to 12% polyacrylamide gels and transferred to nitrocellulose membranes (Protran; Schleicher and Schuell BioScience, Inc). Membranes were blocked in Tris-buffered saline (0.05 M Tris–HCl, pH 7.4, 0.2 M NaCl, and 0.1% Tween-20) containing Tween-20 (0.1%) and nonfat dried skim milk (5% w/v). Membranes were then immunodecorated with rabbit primary antibodies against mouse CREBH, ApoA-IV, apoplipoprotein E, and histone deacetylase diluted at 1:1000 in blocking solution. Signals were developed with 1:7000 dilution of goat anti-rabbit or goat antimouse secondary antibody and visualized with the ProteinSimple system (ProteinSimple).

### Purification of nuclei from mouse liver

Nuclear extracts were prepared from liver as previously described (8). In brief, 300 mg samples of liver tissue were homogenized in 3 ml of buffer containing 10 mM Hepes, pH 7.9, 10 mM KCl, 0.1 mM EGTA, 0.3 M sucrose, 0.5 mM dithiothreitol, 0.74 mM spermidine, and supplemented with protease inhibitors. Homogenates were mixed with 6 ml of cushion buffer (10 mM Hepes, pH 7.9, 0.1 mM EGTA, 2.2 M sucrose, 0.5 mM dithiothreitol, 0.74 mM spermidine, 1 μg/ml aprotinin, and 2 μg/ml leupeptin) and then overlayed with 2 ml of cushion buffer. Following centrifugation at 25,000 rpm for 60 min at 4 °C, the pellet containing nuclei was resuspended by sonication in radioimmunoprecipitation assay buffer for immunoblot analysis.

### Glucose and insulin tolerance tests

Tolerance tests to insulin and glucose were performed as described (41). In brief, 14- to 15-week-old mice treated with doxycycline for 4 to 5 weeks were fasted with free access to water for 6 h (insulin tolerance) or 16 h (glucose tolerance). Blood (<5 μl) was collected from the tail tip prior to and at regular intervals up to 120 min following intraperitoneal injection with 0.25 U insulin/kg body weight (insulin tolerance) or 2 g glucose/kg body weight (glucose tolerance). Blood glucose concentrations were measured using a GE100 Blood Glucose Monitoring System (GE Healthcare).

### Metabolic monitoring

Fourteen-week-old mice treated with doxycycline for 2 weeks were single housed in temperature-controlled cabinets (22 °C) with a 12 h light/dark cycle and monitored using the Promethion Metabolic Screening System (Sable Systems International) at the Metabolic Phenotyping Core (Weill Cornell Medical College). Cage floors were used in place of bedding, and ad libitum access to diet and water was provided. Mice were studied for 72 h at room temperature, where the first 48 h was utilized as an acclimation period, followed by 24 h of data recording (42). Values of VO_2_ and VCO_2_ were determined at 5-min intervals. Values of RER were calculated as VCO_2_/VO_2_. Rates of energy expenditure were calculated from values of VO_2_ and VCO_2_ (42), and physical activities were measured by distances traveled as recorded by sensors that were built into the cages. Values of energy expenditure were calculated and adjusted by analysis of covariance (43) using VassarStats (www.vassarstats.net) to control for differences in lean body mass, which were determined by magnetic resonance spectroscopy (3in1 Body Composition Analyzer; EchoMRI). Food consumption was measured gravimetrically over a 24 h period.

### Plasmids and adenoviruses

Adenovirus encoding mouse nuclear CREBH was generated using the pAdTRACK-CMV shuttle vector system as previously described (44). In brief, mouse nuclear CREBH cDNA was generated by PCR amplification using the cDNA clone (IMAGE: 4211480, BC010786) as a template using the following primers (forward: GATATCCTGGAAAGATGGCGTCCC; reverse: AGATCTCAGGTGCCTGCATGGGCTG). The resulting cDNA was subcloned into the adenoviral shuttle vector pAd-CMV, linearized using the restriction enzyme PmeI, and transformed into AdEasier bacteria containing the adenoviral backbone plasmid pAdEasy-1. As a result, the mouse nuclear CREBH cDNA was recombined into the pAdEasy-1, giving pAdEasy-nuclear CREBH. Adenovirus particles were generated by transfecting human embryonic kidney 293 cells with Pac-I-linearized pAdEasy-nuclear CREBH plasmid using Lipofectamine 2000 (Thermo Fisher Scientific). The virus was then amplified *via* three rounds of human embryonic kidney 293 infection.

### Transcriptomics in primary cultured hepatocytes

Primary hepatocytes cultured from 8- to 10-week-old male wildtype mice were anesthetized with ketamine and xylazine, and prepared as described (44). Livers of wildtype mice were perfused with liver perfusion medium (Life Technologies) for 5 min followed by liver digestion medium (Life Technologies) for 10 min at 5 ml/min. Primary hepatocytes were then cultured with M199 medium supplemented with 1% penicillin/streptomycin and 10% fetal bovine serum in 60 mm dishes at 1 − 10^6^ cells/dish. Cells were transduced with adenoviruses encoding GFP (Ad-GFP) or nuclear CREBH (Ad-nuclear CREBH) at a multiplicity of infection of 100 viral particles/cell. Three 60-mm dish replicates per transduction (Ad-GFP or Ad-nuclear CREBH) were collected 24 h after adenovirus transduction for microarray analysis.

Microarray analysis was performed as described with modifications (32). In brief, total RNA was extracted from primary hepatocytes using QIAzol lysis reagent (Qiagen) followed by total RNA purification using RNeasy Mini columns and on-column RNase-free DNase treatment (Qiagen). Quality of total RNA was determined using the RNA Nano Lab Chip Kit and Bioanalyzer (Agilent). Purified total RNA (100 ng) was labeled using an Ambion WT expression kit (Thermo Fisher Scientific) and hybridized to an Affymetrix Mouse Gene 1.1 ST array plate (Affymetrix). Hybridization, washing, and scanning were carried out on an Affymetrix GeneTitan platform as described by the manufacturer's instructions. Genes were filtered according to expression values >300 and then selected based on a fold change >0.5 (Ad-GFP) or >2 (Ad-CREBH).

### Statistical analysis

Data were analyzed by a mixed model using the fit model procedure of JMP Pro 11.0 statistical software (SAS Institute). For experiments measuring body weight and blood glucose, data were analyzed by a mixed model accounting for genotype (Genotype; *Control*, *Tg-rtTA, Fgf21*^−/−^, or *Tg-rtTA;Fgf21*^−/−^) and time relative to the start of doxycycline treatment. For insulin and glucose tolerance tests, data were analyzed by a mixed model accounting for genotype (genotype; *Control*, *Tg-rtTA*, *Fgf21*^−/−^, or *Tg-rtTA;Fgf21*^−/−^) and time. For glucose tolerance tests involving *Control*, *Tg-rtTA*, *Fgf21*^−/−^, and *Tg-rtTA;Fgf21*^−/−^ mice, the area under the curve was compared by pairwise comparison with Tukey's adjustment. For experiments measuring plasma insulin and FGF21 concentrations, data were analyzed by a mixed model accounting for genotype (genotype; *Control*, *Tg-rtTA*, *Fgf21*^−/−^, and *Tg-rtTA;Fgf21*^−/−^) and time relative to the start of doxycycline treatment. Correlations between plasma Fgf21 and other variables were performed using the fit model procedure of JMP Pro 11.0. All other variables were analyzed by a model accounting for genotype (genotype; *Control versus Tg-rtTA*). For the microarray analysis, data were analyzed using an intensity-based moderated T-statistic, with significance defined at *p* < 0.001 (45).

## Data availability

The microarray dataset has been deposited into the Gene Expression Omnibus database. All other data presented in this article are available upon request.

## Supporting information

This article contains supporting information.

## Conflict of interest

The authors declare that they have no conflicts of interest with the contents of this article.
