# Supplementary material for: Inducible hepatic expression of CREBH mitigates diet-induced obesity, insulin resistance, and hepatic steatosis in mice
Source: J Biol Chem. 2021 May 21;297(1):100815. doi: 10.1016/j.jbc.2021.100815 (PMC8246594; doi:10.1016/j.jbc.2021.100815)
Supplement: Figures S1–S5 and Table S1 [file mmc1.pdf]

**Supporting Information**

# Inducible hepatic expression of CREBH mitigates diet-induced obesity, insulin resistance and hepatic steatosis in mice

Christopher S. Krumm[1], Xu Xu[1], Curtis J. Bare[1], Corey D. Holman[1], Sander Kersten[2], Lukas E. Dow[3], Ann-Hwee Lee[4,5] and David E. Cohen[1,#]

[1]Division of Gastroenterology & Hepatology, Joan & Sanford I. Weill Department of Medicine, Weill Cornell Medical College, New York, NY 10065

[2]Nutrition, Metabolism and, Genomics Group, Division of Human Nutrition and Health, Wageningen University, Wageningen, the Netherlands

[3]Division of Hematology & Medical Oncology, Joan & Sanford I. Weill Department of Medicine, Sandra and Edward Meyer Cancer Center, Weill Cornell Medical College, New York, NY 10065

[4]Department of Pathology & Laboratory Medicine, Weill Cornell Medical College, New York, NY 10065

[5]Present address: Regeneron Pharmaceuticals, Tarrytown, NY, 10591

[#]To whom correspondence should be addressed: David E. Cohen, M.D., Ph.D.; Division of Gastroenterology & Hepatology, Weill Cornell Medical College, Belfer Research Building, 413 East 69th Street, Room 630, New York, NY 10021; Tel: (646) 962-7681; Fax: (646) 962-0427; Email: dcohen@med.cornell.edu.

## Figure S1

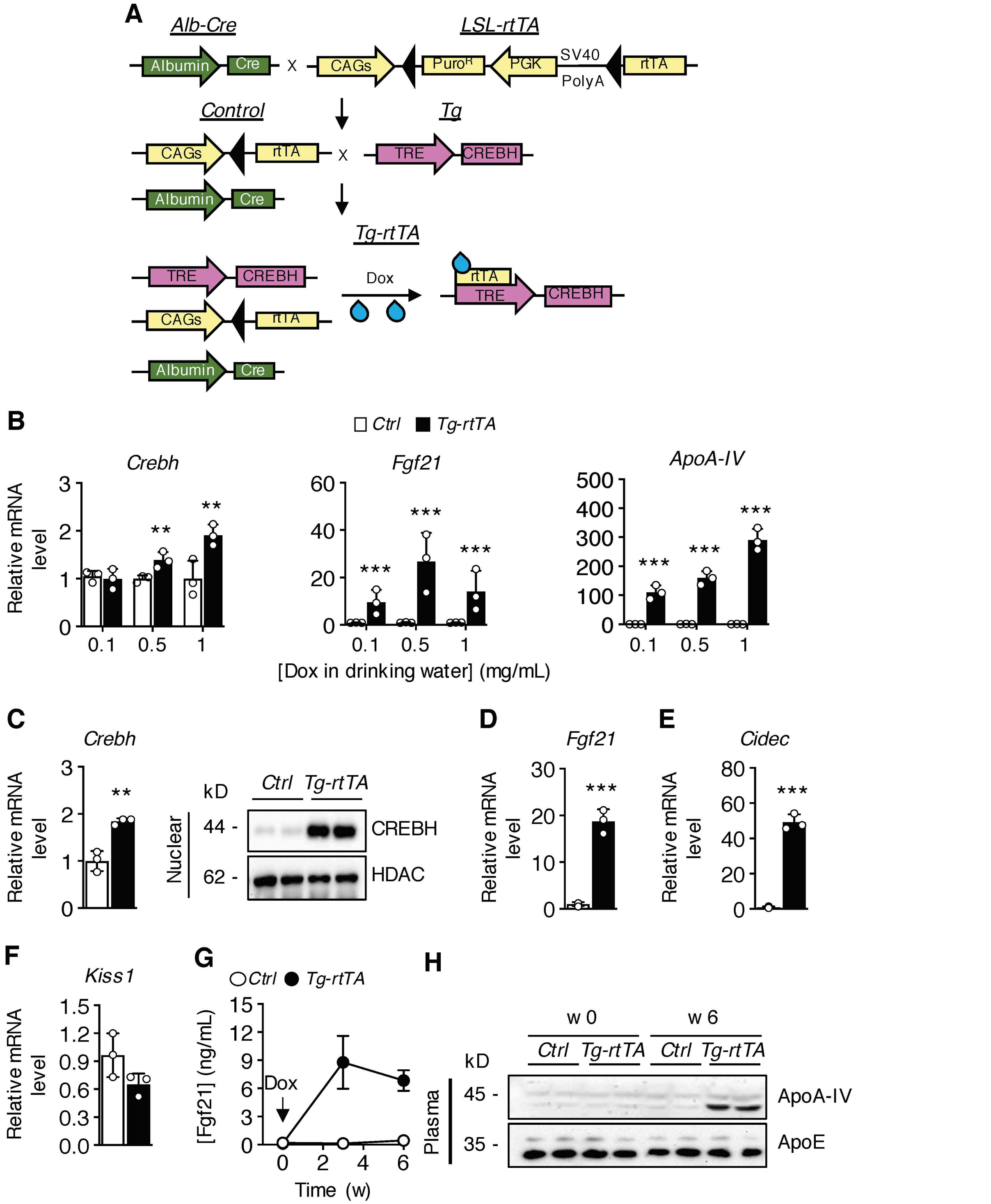


**Supplementary Figure 1: Generation of inducible transgenic hepatocyte-specific nuclear CREBH overexpression (*Tg-rtTA*) mice.** *A*, *Alb-Cre* mice were crossed to *rtTA* mice to generate hepatocyte-specific LSL-rtTA (*Control*) mice. These mice were then crossed with *Tg* containing the nuclear CREBH coding sequence downstream of an inducible tetracycline regulated-element (TRE) to generate *Tg-rtTA* mice. Nuclear CREBH expression was induced in *Tg-rtTA* mice upon treatment with doxycycline (Dox). *B* – H, Six-week old *Control* and *Tg-rtTA* mice were fed a chow diet for 12 weeks and received Dox (1 mg/mL) in their drinking water during the last 6 weeks of dietary feeding. *B*, Relative mRNA expression levels of *Crebh* and associated target genes (*Fgf21* and *ApoA-IV*) in liver. *C*, Relative expression of hepatic *Crebh* mRNA and CREBH nuclear protein, with HDAC utilized to control for unequal loading. *D* – *F*, Relative mRNA expression levels of the CREBH target genes (*D*) *Fgf21*, (*E*) *Cidec* and (*F*) *Kiss1* in liver. (F - G) Plasma concentrations of (*G*) Fgf21 and (*H*) ApoA-IV, with ApoE utilized to control for unequal loading. Arrow indicates the start of Dox treatment. *Control*, n = 3; *Tg-rtTA*, n = 3. Data are means ± SD. **, $p < 0.01$, ***, $p < 0.001$; *Control* vs. *Tg-rtTA*. ApoA-IV, apolipoprotein A-IV; ApoE, apolipoprotein E; Alb-Cre, Cre recombinase driven by the albumin promoter; CAGs, cytomegalovirus enhancer, chicken beta-actin promoter and rabbit beta-globin splice acceptor site; Cidec, cell death-inducing DFFA-like effector C; Fgf21, fibroblast growth factor-21; HDAC, histone deacetylase; Kiss1, KiSS-1 metastasis suppressor; LSL, loxP-flanked polyadenylation signal cassette; rtTA, reverse tetracycline transactivator; *Tg-rtTA*, inducible transgenic hepatocyte-specific tetracycline-regulated element (TRE)-CRE-reverse tetracycline transactivator; TRE, tetracycline-regulated element.

# Figure S2

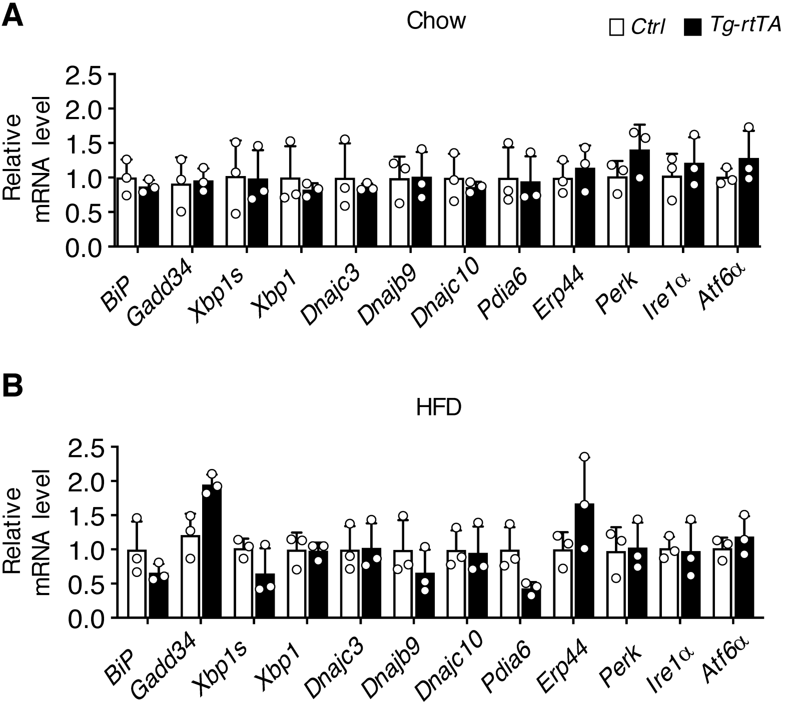


**Supplementary Figure 2: Endoplasmic reticulum stress is not induced in *Tg-rtTA* mice.** Six-week old mice were fed a chow diet or HFD for 12 weeks and received doxycycline (Dox; 1 mg/mL) in their drinking water during the last 6 weeks of dietary feeding. *A – B*, Relative mRNA expression levels of genes that govern endoplasmic reticulum stress (*BiP, Gadd34, Xbp1s, Xbp1, Dnajc3, Dnajb9, Dnajc10, Pdia6, Erp44, Perk, Ire1α, and Atf6α)* in liver extracts of mice fed a (*A*) chow diet or (*B*) HFD were analyzed by quantitative real-time PCR. *Control*, n = 3; *Tg-rtTA*, n = 3. Data are means ± SD. Atf6α, activating transcription factor 6α; BiP, binding immunoglobulin protein; Dnajc3, dnaJ heat shock protein family (Hsp40) member C3; Dnajb9, dnaJ heat shock protein family (Hsp40) member B9; Dnajc10, dnaJ heat shock protein family (Hsp40) member C10; Erp44, endoplasmic reticulum protein 44; Gadd34, growth arrest and DNA damage-inducible protein; HFD, high-fat diet; Perk, PRKR-like endoplasmic reticulum kinase; Pdia6, protein disulfide isomerase associated 6; *Tg-rtTA*; inducible transgenic hepatocyte-specific tetracycline-regulated element (TRE)-CRE-reverse tetracycline transactivator; Xbp1, x-box binding protein 1; Xbp1s, x-box binding protein 1 spliced.

# Figure S3

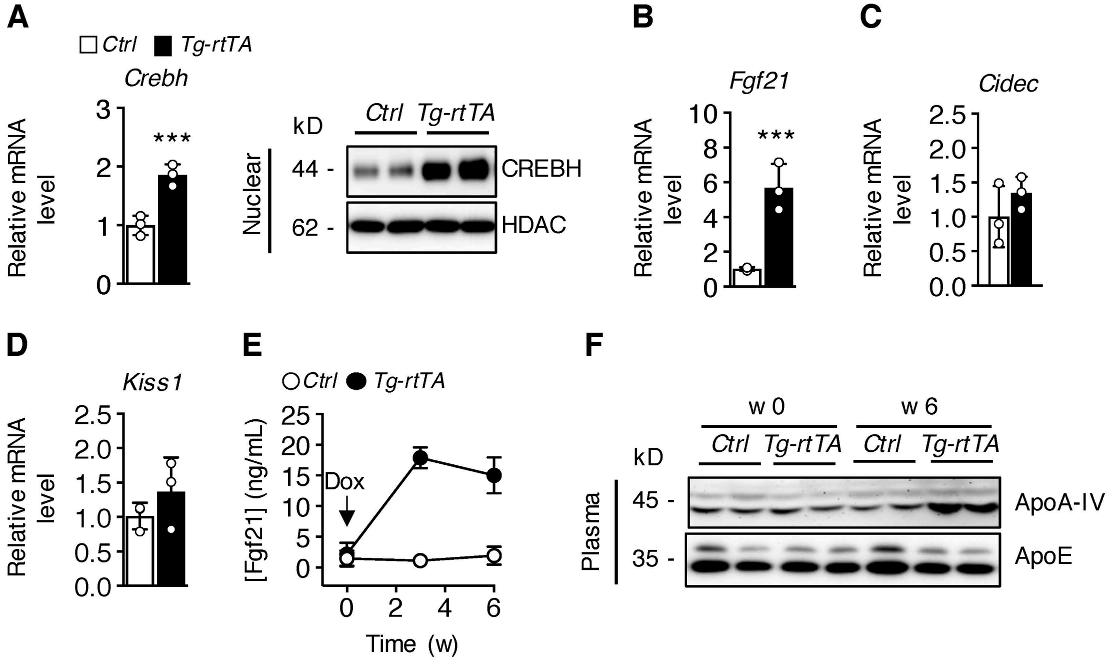


**Supplementary Figure 3: Hepatic overexpression of CREBH in HFD-fed mice.** Six-week old mice were fed HFD for 12 weeks and received doxycycline (Dox; 1 mg/mL) in their drinking water during the last 6 weeks of HFD feeding. *A*, Relative expression of hepatic *Crebh* mRNA and CREBH nuclear protein, with HDAC utilized to control for unequal loading. *B – D*, Relative mRNA expression levels of the CREBH target genes (*B*) *Fgf21*, (*C*) *Cidec* and (*D*) *Kiss1* in liver. Plasma concentrations of (*E*) Fgf21 and (*F*) ApoA-IV, with ApoE utilized as a control for unequal loading. Arrow indicates the start of Dox treatment. *Control*, n = 3; *Tg-rtTA*, n = 3. Data are means ± SD. ***, $P < 0.001$; *Control* vs. *Tg-rtTA*. ApoA-IV, apolipoprotein A-IV; ApoE, apolipoprotein E; Cidec, cell death-inducing DFFA-like effector C; Fgf21, fibroblast growth factor-21; HDAC, histone deacetylase; HFD, high-fat diet; Kiss1, KiSS-1 metastasis suppressor; *Tg-rtTA*; inducible transgenic hepatocyte-specific tetracycline-regulated element (TRE)-CRE-reverse tetracycline transactivator.

# Figure S4

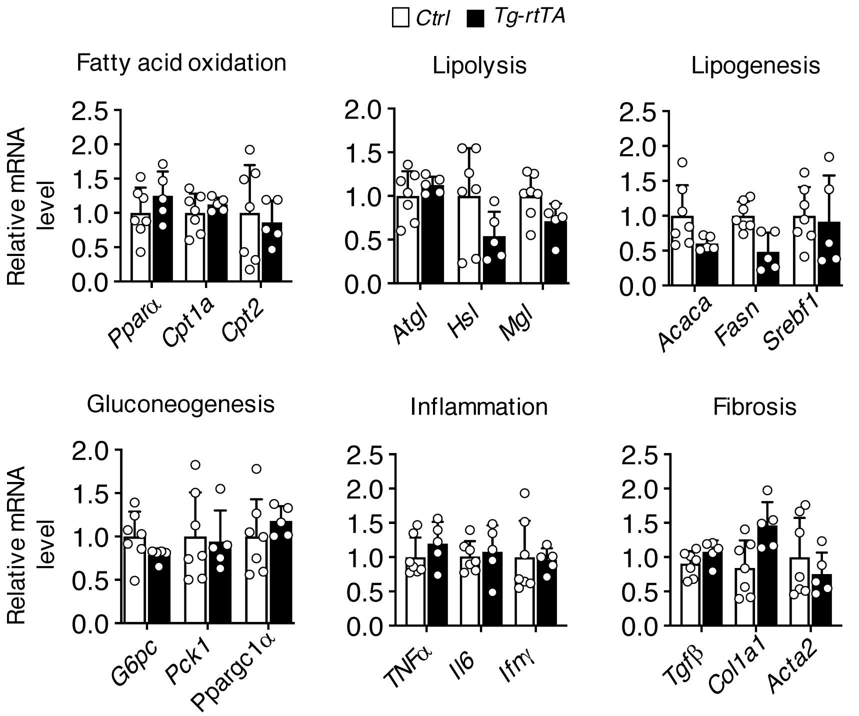


**Supplementary Figure 4: Absence of regulation by CREBH of genes that control fatty acid oxidation, lipolysis, lipogenesis, gluconeogenesis, inflammation or fibrosis in livers of HFD-fed mice.** Six-week old mice were fed HFD for 12 weeks and received doxycycline (Dox; 1 mg/mL) in their drinking water during the last 6 weeks of dietary feeding. Relative mRNA expression levels of genes that govern fatty acid oxidation (*Pparα*, *Ctp1a, and Cpt2*), lipolysis (*Atgl, Hsl, and Mgl*), lipogenesis (*Acaca, Fasn, and Srebf1*), gluconeogenesis (*G6pc, Pck1, and Ppargc1α*), inflammation (*Tnfα, Il6 and Ifnγ),* and fibrosis *(Tgfβ, Col1a1, and Acta2)* in liver. *Control*, n = 7; *Tg-rtTA*, n = 5, Data are mean ± SD. Acaca, acetyl-CoA carboxylase alpha; Acta2, actin alpha 2; Atgl, adipose triglyceride lipase; Col1a1, collagen type 1 alpha 1 chain; Cpt1a, carnitine palmitoyltransferase 1A; Cpt2, carnitine palmitoyltransferase 2; Fasn, fatty acid synthase; G6pc, Glucose-6-phosphatase catalytic subunit ; HFD, high-fat diet; Hsl, hormone-sensitive lipase; Ifnγ, Interferon-γ; Il6, Interleukin 6; Mgl, monoglyceride lipase; Pparα, peroxisome proliferator-activated receptor α; Ppargc1α, Peroxisome proliferator-activated receptor gamma coactivator 1-α; Pck1, phosphoenolpyruvate carboxykinase 1; Srebf1, sterol regulatory element binding transcription factor 1; Tgfβ, Transforming growth factor-β; TNFα, Tumor necrosis factor α; *Tg-rtTA*; inducible transgenic hepatocyte-specific tetracycline-regulated element (TRE)-CRE-reverse tetracycline transactivator.

# Figure S5

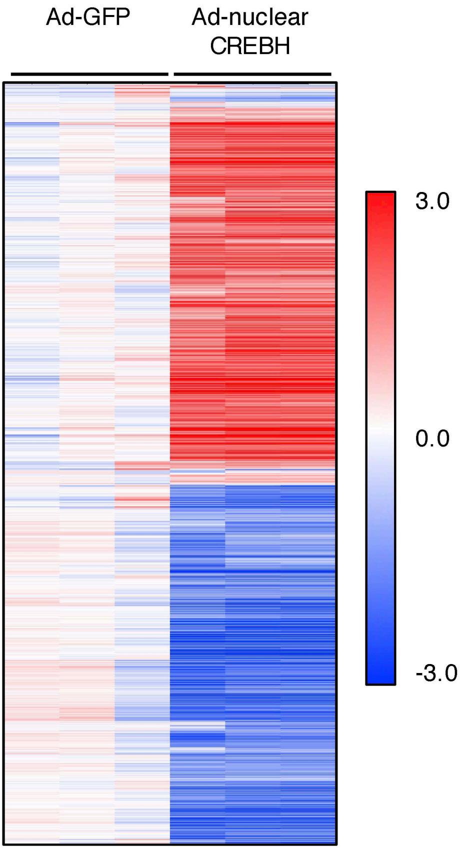


**Supplementary Figure 5: Comparative mRNA expression in primary hepatocytes upon adenovirus-mediated overexpression of nuclear CREBH.** Heat-map showing the fold-changes of genes in primary hepatocytes transduced with Ad-GFP or Ad-nuclear CREBH. Genes that were upregulated (red) or downregulated (blue) by Ad-nuclear CREBH are shown using the criteria of $p < 0.001$ and fold change > 0.5 (Ad-GFP) or > 2 (Ad-nuclear CREBH). The fold-changes shown are signal log ratio. Each column represents an independent sample. Ad-GFP, n = 3; Ad-nuclear CREBH, n = 3. Ad-GFP, adenovirus encoding green fluorescent protein; Ad-nuclear CREBH, adenovirus encoding nuclear CREBH.

**Table S1. Mouse primers used in real-time PCR analysis**

| Gene | NCBI access number | Forward Primer (5' – 3') | Reverse primer (5' - 3') |
|---|---|---|---|
| CREBH | NM_001382818.1 | GGCCATTGACCTGGACATGT | TTCACAGTGAGGTTGAAGCGG |
| Fgf21 | NM_020013.4 | GGAGCTCTCTATGGATCGCCT | TGTAACCGTCCTCCAGCAGC |
| Apoa4 | NM_007468.2 | CCAGCTAAGCAACAATGCCA | TGGAAGAGGGTACTGAGCTGC |
| Apoc2 | NM_001277944.1 | GCATGGATGAGAAACTCAGGG | AAAATGCCTGCGTAAGTGCTC |
| Kiss1 | NM_178260.3 | TGGCAAAAGTGAAGCCTGGA | TCTCTGCATACCGCGATTCC |
| Ucp1 | NM_009463.3 | AGGCTTCCAGTACCATTAGGT | CTGAGTGAGGCAAAGCTGATTT |
| Elovl3 | NM_007703.2 | TTCTCACGCGGGTTAAAAATGG | GAGCAACAGATAGACGACCAC |
| Pparα | NM_011144.6 | CGGAGCTGCAAGATTCAGAAG | CAAAGCGAATTGCATTGTGTG |
| Cpt1a | NM_013495.2 | CTCCGCCTGAGCCATGAAG | CACCAGTGATGATGCCATTCT |
| Cpt2 | NM_009949.2 | CAGCACAGCATCGTACCCA | TCCCAATGCCGTTCTCAAAAT |
| Atgl | NM_001163689.1 | AACACCAGCATCCAGTTCAA | GGTTCAGTAGGCCATTCCTC |
| Hsl | NM_010719.5 | TGTGGCTTGCGCTCTGG | CCGCGCGAACATGACC |
| Mgl | NM_001166251.1 | GGCGAACTCCACAGAATGTT | ACTTGGAAGTCCGACACCAC |
| Acaca | NM_001166251.1 | GAGAGGGCTCAAGTCCTTCC | ACATCCACTTCCACACACGA |
| Fasn | NM_007988.3 | GGAGGTGGTGATAGCCGGTAT | TGGGTAATCCATAGAGCCCAG |
| Srebf1 | NM_001358314.1 | CAGCTCAGAGCCGTGGTGA | TTGATAGAAGACCGGTAGCGC |
| G6pc | NM_008061.4 | CGACTCGCTATCTCCAAGTGA | GTTGAACCAGTCTCCGACCA |
| Pck1 | NM_011044.3 | CTGCATAACGGTCTGGACTTC | CAGCAACTGCCCGTACTCC |
| Ppargc1α | NM_008904.2 | TATGGAGTGACATAGAGTGTGCT | CCACTTCAATCCACCCAGAAAG |
| Tnfα | NM_013693.3 | CCCTCACACTCAGATCATCTTCT | GCTACGACGTGGGCTACAG |
| Il-6 | NM_031168.2 | TAGTCCTTCCTACCCCAATTTCC | TTGGTCCTTAGCCACTCCTTC |
| Ifnγ | NM_008337.4 | TGCTGAGTCCGCAGCAGG | CATCAGAGTCCATGGGGAGA |
| Tgfβ | NM_011577.2 | CCAGCGTTCCAGCAAAAACA | CTGGAGCTGAACCACGGTAG |
| Col1a1 | NM_007742.4 | TGTCAGCACCATCTCTGATGA | CTGCAGAGAGCAGGGGAG |
| Acta2 | NM_007392.3 | GCGTGGCTACAGCTTCACCC | CTTGATGTCACGGACGATTTC |
| BiP | NM_001163434.1 | TCATCGGACGCACTTGGAA | CAACCACCTTGAATGGCAAGA |
| Gadd34 | NM_008654.2 | GAGGGACGCCCACAACTTC | TTACCAGAGACAGGGGTAGGT |
| Xbp1 | NM_013842.3 | CCTGAGCCCGGAGGAGAA | CTCGAGCAGTCTGCGCTG |
| Xbp1s | NM_001271730.1 | GACAGAGAGTCAAACTAACGTGG | GTCCAGCAGGCAAGAAGGT |
| Dnajc3 | NM_008929.3 | GGCGCTGAGTGTGGAGTAAAT | GCGTGAAACTGTGATAAGGCG |
| Dnajb9 | NM_013760.4 | TAAAAGCCCTGATGCTGAAGC | TCCGACTATTGGCATCCGA |
| Dnajc10 | NM_024181.2 | GCTGGCTGATCACATTCTGCT | GCCGTCCAACATGCCACTA |

**Table S1. Mouse primers used in real-time PCR analysis**

| Gene | NCBI access number | Forward Primer (5' – 3') | Reverse primer (5' - 3') |
|---|---|---|---|
| Pdia6 | NM_027959.4 | CTAGCAGTCAGCGGTCTGTAT | CACAGGCCGTCACTCTGAA |
| Erp44 | NM_029572.3 | TGCATCCAATTTTGAGGAAGCA | CTTATCCTGTACCTCTGGGCTAT |
| Perk | NM_010121.3 | TGTCGCCAATGGGATAGTGACGAA | AATCCGGCTCTCGTTTCCATGTCT |
| Ire1α | NM_023913.2 | GCAACCATCCTTTTGGCAAAT | AACAGTCAAGGTTGCAGGCG |
| Atf6α | XM_030253420.1 | CAAGATCAAGCCCCACCTGAT | AGTTCGCCCCAACCAGTACTT |
| Cidec | NM_001301295.1 | ATGGACTACGCCATGAAGTCT | CGGTGCTAACACGACAGGG |
| Actβ | NM_007393.5 | TACCACCATGTACCCAGGCA | CTCAGGAGGAGCAATGATCTTGAT |
